# Reusable building blocks in biological systems

**DOI:** 10.1098/rsif.2018.0595

**Published:** 2018-12-19

**Authors:** Victor Mireles, Tim O. F. Conrad

**Affiliations:** 1 Department of Mathematics and Computer Science, Freie Universität Berlin, Berlin, Germany; 2 International Max Planck Research School for Computational Biology and Scientific Computing, Max Planck Institute for Molecular Genetics, Berlin, Germany

**Keywords:** module sizes, building blocks, near decomposability, evolution of modularity, modularity

## Abstract

One of the most widely recognized features of biological systems is their modularity. The modules that constitute biological systems are said to be *redeployed and combined* across several conditions, thus acting as building blocks. In this work, we analyse to what extent are these building blocks reusable as compared with those found in randomized versions of a system. We develop a notion of decompositions of systems into phenotypic building blocks, which allows them to overlap while maximizing the number of times a building block is reused across several conditions. Different biological systems present building blocks whose reusability ranges from single use (e.g. condition specific) to constitutive, although their average reusability is not always higher than random equivalents of the system. These decompositions reveal a distinct distribution of building block sizes in real biological systems. This distribution stems, in part, from the peculiar usage pattern of the elements of biological systems, and constitutes a new angle to study the evolution of modularity.

## 1. Introduction

In many biological systems, one can identify *sets of elements that act together in performing some discrete physiological function* [1], which have been called functional modules. These modules can be, for example, genes that form a signalling pathway, enzymes involved in a metabolic pathway, or microbial species that co-occur in different ecosystems [2]. Furthermore, it has been suggested that biological processes can be described in terms of modules [3]. In other words, the set of elements involved in a given process is the union of some collection of modules that act as building blocks. For example, the genes active in yeast during the hypo-osmotic shift are those regulated by Cmk1 plus those regulated by Pbt1 [4]. The notion of modularity has been further developed to include a hierarchical organization of modules [5,6], overlapping modules [7] or a dynamic membership of elements into modules [8], ultimately yielding an intricate characterization of biological complexity.

The consequences that such a modular organization has for biological systems have been studied from many standpoints [9,10]. From an evolutionary angle, modularity has been linked to evolvability [11] and robustness [12], and evolutionary conserved modules have been studied in several taxa (e.g. [13,14]). From a physiological point of view, functional modules have been associated with responses to changing environments [15] and are thought to be determined, at least in part, by regulatory mechanisms [16], often coupled to physical processes affecting cells [17].

In general, modules are thought to exhibit at least two properties: independence from one another, and reusability across different scenarios or conditions.

Independence of modules from each other [18] means that the elements constituting one module interact more among themselves than with those constituting another module. After fixing the set of elements one is dealing with (e.g. genes, traits or species), there are many choices for the exact definition of *interaction*, each leading to different types of modules: functional, evolutionary, variational, developmental, etc. For in-depth discussions of these definitions, the reader can refer to [10] and references therein. Independence enables groups of elements to vary independently, without altering, in a countervailing fashion, other characteristics of the organism [19]. That is, modularity is a means for reducing pleiotropic effects of genes, which, in turn, increases evolvability [11].

Reusability is the quality of modules of being *redeployed and combined* [20] across several conditions, playing the role of reusable *building blocks* [21,22]. Just as genes can be co-opted [23] to perform novel functions, sets of genes have also been documented as having multiple uses, perhaps the most famous case being the sonic hedgehog signalling pathway. When a mechanistic description of the interactions among elements is not known, the reusability of a set of elements is often enough to consider it a putative building block, as in the case of co-expression modules [24]. The focus of this work is the role of modules as building blocks and their reusability.

Reusability is mediated by several properties of biological systems, such as the combinatorial nature of transcription factor regulation [25], the different tissue specificities that interactions of a given protein can have [26], or the multifunctional nature of gene circuits [27].

The reuse of biological modules leads to an increase in phenotypic variation by loosening the dependence on genotypic variation [28]. This is achieved by two pleiotropic mechanisms, whose potentially deleterious effects are limited by the independence of modules. The first mechanism magnifies the variations in the loci encoding elements within modules. If a module is reused in several conditions, the effects of these variations are pleiotropic because they appear under all of these conditions. The second mechanism magnifies the variations in the loci that determine the reuse of a particular module. If this reuse is increased by such a variation, all the processes within the module, as well as its interactions with elements outside of it, will be available at once under a new set of conditions. This is pleiotropic because modules are not completely independent and thus these intermodular interactions are multiple. This second mechanism leads to the notion of modules as building blocks that are combined verbatim into different phenotypes.

Descriptions of biological systems in terms of building blocks are shorter than those in terms of their individual components (in the Kolmogorov complexity sense), and this reduction in description length increases with reusability. In this context, a proposed building block can range from a high reusability building block, providing parsimonious descriptions of the observed phenotypes [29], to a single use, ad hoc building block that is employed in a single condition. While reusable building blocks have been widely identified in biological systems, it is not clear if these are the only systems which exhibit them, or if they do so in some distinctive fashion. In this work, we aim at quantitatively comparing the reusability of the building blocks present in biological systems with that of those present in random systems.

The reusability of building blocks is related to their size. Smaller ones can, in principle, be more reusable because very small sets of elements (e.g. singletons) are more likely to be entirely present in many conditions than very large sets. While this relationship between size and reusability does not always hold, studying the building block size distribution in biological systems is a good starting point for studying the reusability of their building blocks.

In general, the study of module size distributions has proven interesting from several standpoints. On the one hand, as the work related to the size distribution of the paralogue gene [30] and protein [31] families shows, it can aid in developing models for the evolution of sets of biological elements. In this sense, the understanding of the evolution of modularity, which is still a topic of debate (e.g. [6,32,33]), can be aided by studying the distribution of module sizes. On the other hand, finding estimates of the distribution of module sizes can aid in the calibration of several module-identifying algorithms (e.g. [34–36]) which have parameters that influence which sizes of modules they can detect (as discussed, for example, in [37]). Finally, as discussed in [38], knowing the module size distribution can improve the null models used for gene set enrichment analyses. We believe these advantages to hold also in the particular case of modules being studied in their capacity as building blocks.

## 2. Phenotypic building blocks

We wish to exclude from the discussion of this paper any preexisting notions stemming from any of the many definitions of modularity available in the literature. In particular, since this work focuses on the property of modules of being reusable across phenotypes, we wish to set aside discussions regarding their evolutionary origin or the mechanistic relationships between their constituents. Therefore, we will build upon an abstract notion of module which we call a *phenotypic building block* (PBB). This notion aims at capturing the building block role of modules, with respect to the phenotypes a system can exhibit under different conditions. PBBs are thus derived from the observation of a set of phenotypes, and their capacity as building blocks is only with respect to these. In other words, the only claim made is that PBBs *build* the observed phenotypes, without any further assumption as to the underlying mechanisms. We now informally describe this notion, but the reader is referred to appendix A for a concise definition, and proofs of all the claims made in this section.

Consider a system made up of a fixed set of elements which expresses different phenotypes. An example of such a system is the collection of genes in an individual, each of which is expressed differently in different tissues, or under different conditions. A PBB is a set of elements that is employed as a whole, in combination with other such blocks, to form the set of elements present in a set of phenotypes.

**Figure 1.**
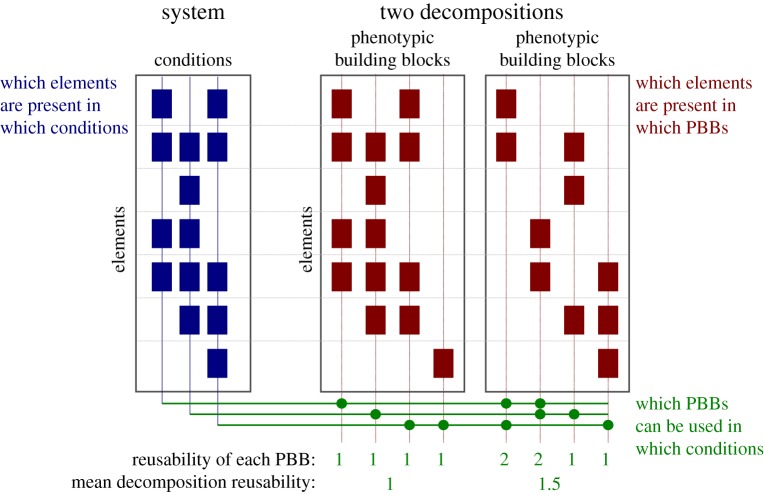
Different decompositions have different reusabilities. Two different decompositions of a given system can have building blocks with varying degrees of reusability (number of conditions using each building block). On the left, a system consisting of seven elements is observed across three conditions: the blue boxes represent which elements are present in which conditions. On the right, two different decompositions of the system into *k* = 4 building blocks are shown. The dots below indicate which building blocks are used in which conditions. For example, the elements present in the right-most condition are the union of those present in the last two building blocks of the first decomposition, or the first and last of the second decomposition.

In this scenario, the different phenotypes can be decomposed as the union of a set of such PBBs. After performing such a decomposition, one can speak of the *reusability* of a PBB: the number of different phenotypes in which it is employed. It can be proven that, given a set of observations of such a system across several phenotypes, there are many possible ways to decompose it into PBBs, as illustrated in figure 1. However, it is possible to find a decomposition in which the average reusability of its PBBs is maximal, and these we call *k*-maximally reusable decompositions (*k*-MRD), when they are made up of *k* PBBs.

We must note that the reusability of a *k*-MRD increases as *k*, the number of PBBs, increases. However, this increase in reusability is not the same for all systems. Furthermore, the relationship that *k* and the reusability of *k*-MRDs hold in a particular system is inherent to properties of the system itself, such as the frequency with which each element is used across all conditions, or the total number of different presence/absence profiles of elements. In what follows, we study both random and biological systems, and shed some light on the relationship between *k* and the reusability of their *k*-MRDs. We are specifically interested in (i) seeing what part of this relationship is consistent across different systems and (ii) seeing how different this relationship is in real systems as opposed to random ones.

## 3. Data from biological systems and their random equivalents

In this work, we have studied biological systems of two kinds: protein expression profiles across tissues and miRNA expression across different conditions. Furthermore, for each of them, we have created randomized versions in order to assess the relevance of the reusability of their PPBs.

The presence and absence of proteins in different tissues of one organism is a particularly relatable example of a fixed repertoire of elements (the proteins encoded in the genome) being deployed in different combinations and leading to different functionalities. A detailed investigation of these presence and absence patterns was carried out in the work of Souiai *et al.* [39], who focused on the interactions between proteins. Here, we complement that work by providing an analysis of the PBBs that could be used to describe the different phenotypes studied, with particular attention to their reusability. We do so using the same data. The data describing these different presence/absence patterns are obtained by equating the presence of a single expressed sequence tag (EST) with the presence of the protein encoded by the corresponding gene. As discussed in [40], EST data are at least as good as those produced by other technologies for quantifying the presence/absence of genes.

The second type of data we use is miRNA expression data, as measured by quantitative reverse transcription–polymerase chain reaction (RT-PCR) [41]. The regulation of miRNA is influenced by both gene regulation and external chemical stimuli [42], thus making miRNA presence/absence patterns a reflection of both endogenous and exogenous factors. Importantly, miRNA expression data have the advantage of being small enough that one can produce and analyse several replicates of the random equivalents of them. We use the datasets that are listed as using the platform GPK13987 in the Gene Expression Omnibus [43].^1^ For these datasets, a threshold of 35 PCR cycles without detection was used to consider a miRNA not present in a condition. We tested with values for this threshold between 25 and 35 and found no difference in the results shown here.

In order to characterize biological systems in terms of the obtained bounds on reusability, we compare with decompositions of two types of randomized equivalents of the real matrices studied, which we briefly describe here and in detail in appendix B. The first type are random binary matrices such that the number of elements active in every condition remains the same as in the real matrix, but the identity of these elements is randomized. We call these density-preserving random matrices (DP-Rand).

The second type of randomized matrices preserve the distribution of *element usage*, that is, there is the same number of condition-specific elements, the same number of elements active in two conditions and so on. Element usage is also known as expression breadth [40]. We call this second type row sum sequence-preserving random matrices (RSS-Rand). We use these kind of matrices because the element usage of all observed datasets greatly differs from the binomial one expected for DP-Rand matrices (see figure 5 and electronic supplementary material, figure S1). This observed element-usage distribution exhibits a great number of constitutive elements, and thus enforces the existence of very large, very reusable PBBs.

**Figure 2.**
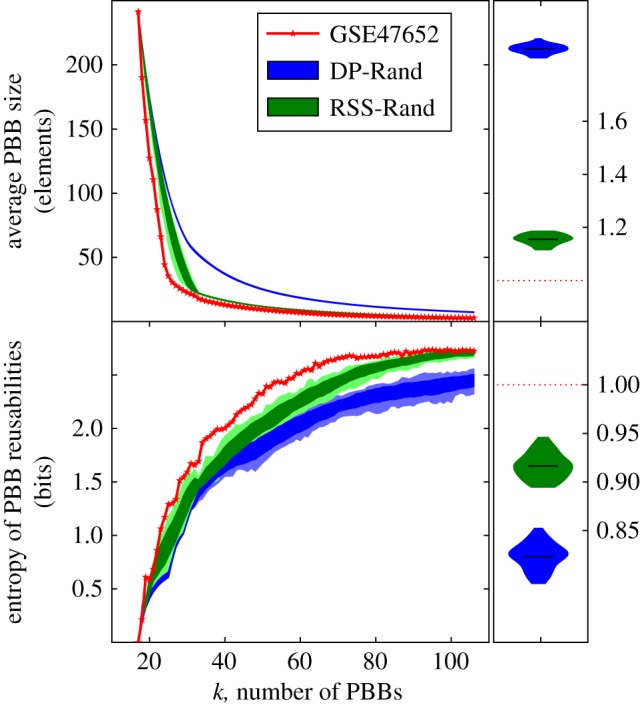
Mean size and reusability entropy of *k*-MRDs of real and randomized systems. Biological systems can be decomposed into smaller and more uniformly reusable PBBs than random equivalent systems, regardless of the number of PBBs. Top left: an example of how the average PBB size of *k*-MRDs changes with the number of PBBs (*k*) for a miRNA expression dataset (GSE 47652) and 100 randomized versions of it: 50 that preserve the column density (blue) and 50 that preserve the row sum distribution (green). The average module sizes of the *k*-MRDs of these random versions are within the ranges shown in the light shaded regions, and the dark shaded regions contain one standard deviation around the mean. Top right: the ratios of area under the curve (AUC) between the red curve and each of the curves corresponding to the randomized systems are all greater than 1, which summarizes that *k*-MRDs of real systems are made of smaller PBBs. The ratio between two AUCs is equivalent to the ratio of two averages. Bottom left: for all possible *k*, the entropy of the distribution of PBB reusability was computed for the same miRNA expression dataset and its 100 random equivalents. A low entropy implies all PBBs have the same reusability. The shaded regions show the range (light) and one standard deviation around the mean (dark) of the entropies of PBB sizes for DP-Rand (blue) and RSS-Rand (green) random equivalents of the system. Bottom right: the ratio of AUCs of PBB reusability entropies is below 1, indicating higher reusability entropy for the real system. (Online version in colour.)

For a given dataset, real or random, *k*-MRDs are computed for all possible values of *k*, and for each of them three quantities are extracted: mean PBB size, maximum PBB size and entropy of the PBB reusability distribution. This last quantity measures how uniformly reusable the PBBs of a decomposition are: it is low if all PBBs have the same reusability, and high if reusabilities are uniformly distributed. In order to compare real datasets and their randomized equivalents using one of these quantities, we measure the average, over *k*, ratio between the quantity in the randomized dataset and the quantity in the real dataset (e.g. figure 2).

## 4. Results

**Figure 3.**
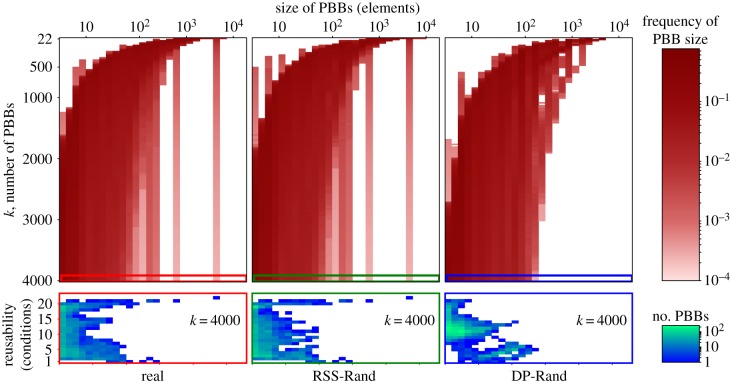
PBB size distributions and size/reusability relationship. When decomposed into maximally reusable decompositions of *k* PBBs (*k*-MRDs), biological systems have a wider range of PBB sizes, and more large and highly reusable PBBs than random equivalents of the DP-Rand type; these features are recovered in RSS-Rand equivalents. The expression data on human tissues [39] and two random equivalents of it (shown in different columns) were decomposed into *k*-MRDs consisting of between 22 and 4000 PBBs. Top: as the number of PBBs increases, their average size decreases for both real and random systems. Yet, the real system always exhibits few very large PBBs, as well as more very small PBBs than a totally random system. Bottom: an example is shown for *k* = 4000 of how much are PBBs of different sizes reused. In this case, the real system has more large and very reusable PBBs, as well as small and condition-specific PBBs, than the DP-Rand system. (Online version in colour.)

**Figure 4.**
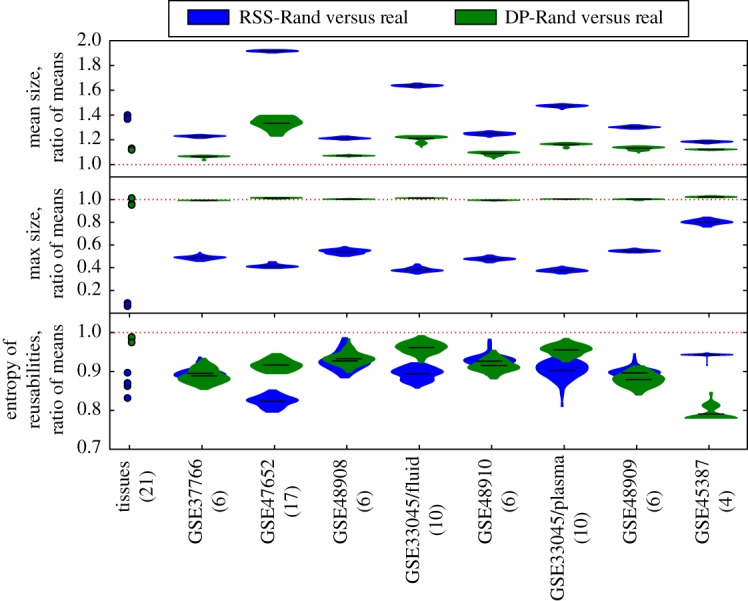
Difference between *k*-MRD of all datasets and their random equivalents. *k*-MRDs of all datasets studied exhibit both smaller average PBB size and larger maximum PBB size than random equivalents of the system. Each system and its random equivalents were decomposed into *k*-MRDs of all possible numbers of PBBs and, for each, three quantities were computed: the average PBB size (top), maximum PBB size (middle) and entropy of the PBB reusability distribution (bottom). Shown are the distributions of the ratios between the average of each quantity in a randomized system, and the average in the real system, as shown in figure 2. While mean and maximum PBB sizes can be replicated by random systems with the same row sum distribution as the real system (RSS-Rand), the same cannot be said of the distribution of PBB reusabilities. Shown in parentheses are the number of conditions in which each system was observed.

**Figure 5.**
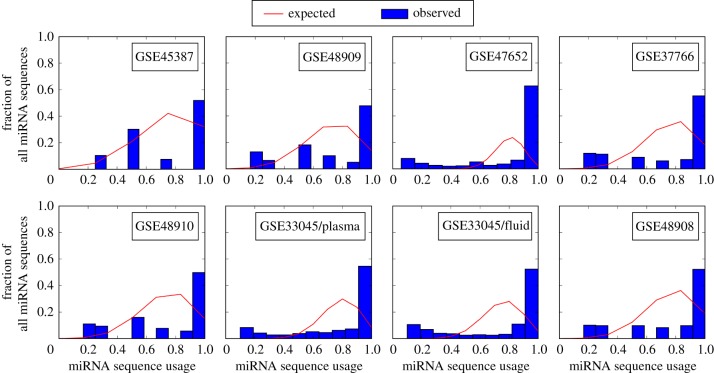
Element usage distribution in miRNA datasets. The distribution of element usage in biological systems promotes big and reusable sets of elements. For several miRNA expression datasets (see text), the distribution of usage of elements (different miRNA sequences), measured by the number of conditions that contain each element, is different from the binomial distribution (line) that would correspond to the elements being distributed randomly across the conditions while preserving the total number of condition/element occurrences (i.e. the density of matrix *C*). (Online version in colour.)

The PBBs that constitute *k*-MRDs are, on average, smaller in biological systems than in their random equivalents, while, simultaneously, the maximum PBB size is larger (see figure 2 for an example using a miRNA expression dataset and figure 3 for an example using the protein expression data). For the average PBB size to remain low in the presence of such large PBBs, the rest of the PBBs must be very small. While the maximum PBB size is a direct consequence of the element usage distribution, and can thus be replicated by the RSS-Rand equivalents of a system, the same cannot be said of the mean PBB size (figure 4 top and middle). We should note that the element usage is markedly different from the expected (binomial) distributions of row sums of a random matrix with the same density (figure 5).

A smaller PBB size implies, for any fixed number of PBBs, a smaller overlap between them. Therefore, these results imply that biological systems can be decomposed into less overlapping, more independent PBBs than random systems, which is a corroboration of the near-decomposability [44] property of natural systems. That being said, the fact that mean PBB size is more similar between real systems and their RSS-Rand equivalents than between real systems and their DP-Rand equivalents suggests that some part of this near decomposability could be due to the element usage distribution.

**Figure 6.**
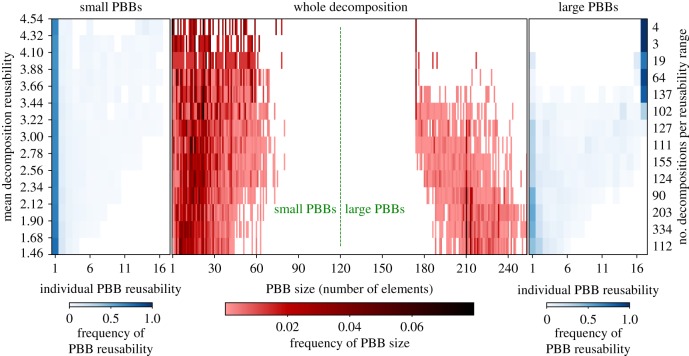
The bimodality of the distribution of sizes of PBBs is not exclusive to *k*-MRDs, but the reusability of large modules is. In total, 1529 decompositions were obtained on the GSE47652 dataset, most of which were far from maximally reusable. Each consists of *k* = 26 PBBs of different sizes. On the vertical axis are different ranges of mean decomposition reusability (number of decompositions in each range, shown on the right). The centre figure shows the size distribution of the PBBs in decompositions of different mean reusabilities. Also shown is the reusability of individual PBBs, after being separated into *small* (left) and *large* (right). The separation was chosen at size 120, which divides the two modes of the size distribution. Only decompositions that are close to optimal exhibit the large, highly reusable PBBs.

Even though these decompositions are maximally reusable, real biological systems have PBBs of a wider range of reusabilities, as opposed to random systems (figure 4 bottom). Having PBBs of more uniformly distributed reusabilities implies the presence of both condition-specific PBBs and constitutive or almost constitutive PBBs. The latter kind of PBBs is also the largest in all of the real systems analysed here (see figure 3 top for an example). If one analyses decompositions which are not maximally reusable, the existence of large PBBs is preserved, but these are not necessarily the most reusable ones (see figure 6 for an example on a miRNA expression dataset for *k* = 26).

It is important to mention that, of the nine systems studied, four had average reusabilities close (within one standard deviation of the mean) to the ones exhibited by their DP-Rand equivalents, and one of them had an average reusability close to the ones exhibited by its RSS-Rand equivalents. Average reusability, therefore, cannot be said to be characteristically high in biological systems.

**Figure 7.**
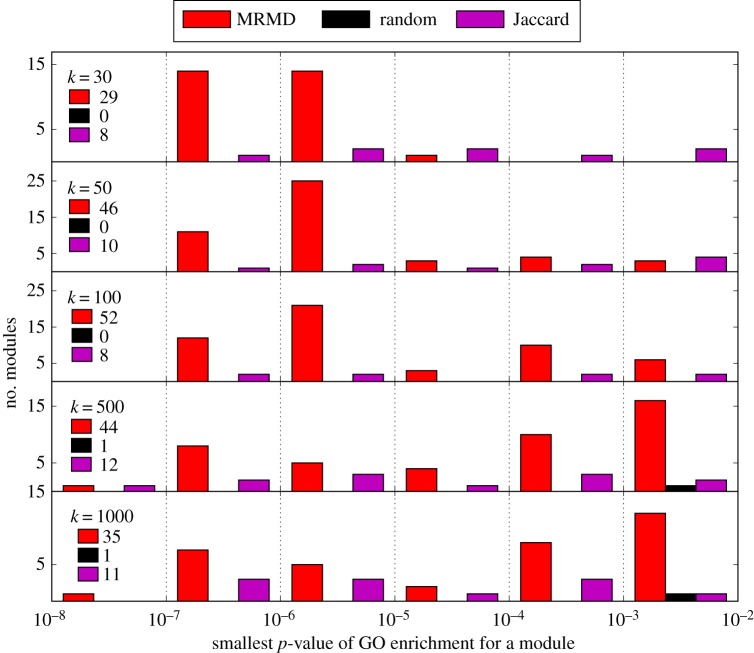
Gene ontology (GO) term enrichment analysis of PBBs in *k*-MRDs. *k*-MRDs have more PBBs which are significantly enriched for GO terms than agglomerative clusterings or randomly chosen PBBs with equivalent sizes. Using the data in [39], and for various values of *k* each of three types of groupings of proteins into PBBs was performed: *k*-MRD, agglomerative clustering with Jaccard coefficient as similarity metric, and random grouping into *k* PBBs of the same sizes as those in the *k*-MRD. For each obtained PBB, the *p*-value of its enrichment (Fisher test, implemented in [45]) to the most-enriched-for GO term was computed and a histogram of these *p*-values was made. The histogram only shows those enrichments with *p* < 0.01 after Bonferroni correction for both the number of GO terms and the number of PBBs. For each value of *k*, the total number of PBBs each method returns with an enrichment with a *p*-value < 0.01 is also shown. (Online version in colour.)

Analysing the presence/absence of proteins in 21 human tissues using the data from [39], we find that several of the PBBs found in *k*-MRDs are functionally relevant (figure 7). Specifically, for a wide range of *k*, *k*-MRDs include more PBBs which are significantly (*p* < 0.01 after Bonferroni correction for multiple testing) enriched for gene ontology terms than those found using agglomerative clustering based on Jaccard distances (a commonly used method that, in the case of binary expression, guarantees proteins grouped together are co-expressed in the greatest possible number of tissues). This is despite the fact that the criteria for finding *k*-MRDs is simply to maximize reusability, without including any additional biological information.

## 5. Discussion

Within the framework presented here, any system can be decomposed into PBBs. PBBs represent reusable modules which can be *redeployed and combined across different conditions* [20]. However, these redeployments can differ across systems and identifying these differences can serve as a way to compare both systems and collections of conditions. Since reusability is often mentioned as a property of biological modules, it would be desirable to understand in which sense is this reusability characteristic of biological systems.

Let us recall that, for a given *k*, the *k*-MRD is just one in many decompositions of a system into *k* PBBs. Since the criterion for finding *k*-MRDs is to maximize reusability, and no other biological information is taken into account, we cannot make any claim about their biological relevance. However, their mean reusability is, by definition, an upper bound on the mean module reusability of any decomposition into *k* modules, in particular any whose modules are in some sense biologically relevant.

While high average reusability of their PBBs does not seem to be a defining feature of biological systems, the uniformity of the distribution of PBB reusabilities, as quantified by its Shannon entropy, does seem capable of distinguishing real biological systems from at least two kinds of random systems. This uniformity in the distribution is greatly influenced by the presence of large constitutive or almost constitutive PBBs, which seems to be a hallmark of biological systems.

The intuition that reusability of a PBB is anticorrelated with its size is wrong in the case of biological systems. On the one hand, these systems exhibit very large constitutive PBBs. On the other, even when these systems are decomposed into very small PBBs some of them are condition specific. These particular distributions of PBB sizes and reusabilities hint at bounds on the processes shaping the modular organization of biological systems. For example, if one adopts the theory that modules have evolved as a response to changing but recurrent environments [15], these distributions could shed some light on the magnitude and frequency of these changes. See appendix C for a brief discussion.

Interestingly, the particular PBB size distribution exhibited by the real systems analysed is approximated by random systems in which element usage is the same as in the real system. PBB size distribution conveys information about the near decomposability of a system, since, for a fixed number of PBBs, larger average PBB size implies more overlap among them, which in turn implies less independence. It should also be noted that the distribution of PBB size found in biological systems is not only present in *k*-MRDs. Indeed, less reusable decompositions also exhibit many small and some very large PBBs (although the latter are not necessarily highly reusable). These two facts suggest that part of the observed independence of biological modules could be due to the peculiar element usage distributions found in nature: one in which both seldom used and always used elements are overrepresented.

This particular U-shaped element usage distribution, or expression breadth, has been reported in humans [40] and mice [46], and is also present in all the species studied in [47] (electronic supplementary material, figure S1). There is evidence that similar usage distributions are present if one considers the presence/absence of genes across species [48], as well as in artificial systems [49], where they have been related to the overall frequency of components [50]. While there are many studies regarding the adaptive nature of modularity, there are, to our knowledge, no studies on the fitness of distributions of individual element usage. On the contrary, non-adaptive explanations for the U-shaped distributions of genes across species have been put forward [51,52] which suggest that drift is responsible for genes being present in few genomes while selection imposes genes present in many.

Studying the relationship between element usage distribution and modularity can aid not only in understanding the evolutionary origins of the latter. It can also serve as a tool for the assessment of the significance of any putative module or sets of modules. In the field of ecological interactions, it was long ago recognized that any identification of communities should be considered against the backdrop of a null model which takes into account the column and row sums of presence/absence matrices [53]. We believe that the results shown here highlight the need for such a null model for biological modularity which takes into account, among other things, module size and element usage distributions.

## Supplementary Material

Gene usage distribution across mammalian and chicken organs

## Endnotes

1 Those with accession nos. GSE37766, GSE48910, GSE48909, GSE48908, GSE47652, GSE45387 and GSE33045 (divided into fluid and plasma subsets).

## Data accessibility

All data used in this work are publicly available in the cited sources: Gene Expression Omnibus [43],^2^ and electronic supplementary materials of [39,47]. An implementation of the algorithm used for decompositions can be found at https://github.com/syats/ModuleReusability.

## Appendix A. Definition of phenotypic building blocks and decompositions

We consider a system consisting of *m* different elements, or units $U=\{u_{1},u_{2},\ldots ,u_{m}\}$. This system is observed under *n* different conditions. From these observations, we derive a presence/absence matrix *C* ∈ {0, 1}^*m*×*n*^. The set of elements active in the *i*th condition is denoted by *c*_*i*_, and corresponds to the set of non-zero entries of the *i*th column of matrix *C*.

A *phenotypic building block (PBB)* is a set *b* ⊂ *U*, and a decomposition is a set of PBBs $\left\{b_{1},b_{2},\;\ldots \;b_{k}\right\}$ such that for i∈{1,2,…,n} we have that $c_{i}=\cup_{\;j\in s_{i}}b_{j}$ for some set of indices $s_{i}\subset \{1,2,\ldots ,k\}$ . A set of PBBs can also be represented by an *indicator* matrix *B* ∈ {0, 1}^*m*×*k*^ such that *B*[*x*, *j*] = 1 if and only if *u*_*x*_ ∈ *b*_*j*_. Matrix *B* represents a decomposition into *k* PBBs if and only if there exists a matrix *S*(*B*) ∈ {0, 1}^*k*×*n*^ such that *C* = *σ*(*B* *S*(*B*)), where σ is the signum function, equal to 1 if its argument is positive and 0 otherwise. The reusability of a PBB *b*_*j*_ is given by $\sum_{i}S(B)[j,i]$, the number of conditions *i* for which *b*_*j*_ ⊂ *c*_*i*_. The reusability of a decomposition is the average reusability of its PBBs, which can be computed by

$$R(B)=\frac{1}{k}1_{k}^{T}S(B)1_{n},$$

where $1_{l}$ is an all-ones vector of size *l*.

We make two additional assumptions: (1) that the set of elements active in one condition cannot be a subset of those active in another and (2) that *n* ≤ *k* ≤ *m*. Assumption 1 is done without loss of generality: consider a set of conditions encoded by a matrix *C* such that $c_{i_{1}}\subset c_{i_{2}}$. We can then build a matrix *C*′ that is identical to *C* except it lacks the *i*_2_’th column and in its place has a column with ones for the elements of $c_{i_{2}}\setminus c_{i_{1}}$. *C*′ satisfies assumption 1 and a decomposition of it is also a decomposition for *C*. We assume the first inequality in assumption 2 because we want decompositions to be exact, that is, ‖*C* − *σ*(*B* *S*(*B*))‖ = 0. The second inequality of assumption 2 is because a collection of singleton PBBs are, trivially, a decomposition of *C* ($C=I_{m}C$ for $I_{m}$ the *m* × *m* identity matrix). Within this range, there is always at least one decomposition for each value of *k*.

**A.1. Finding decompositions**

Given the presence/absence matrix *C* and *k*, a number of PBBs, there are, in general, many possible decompositions of *C* into *k* PBBs, even after accounting for permutations of the columns of *B* (figure 1). In this work, we are particularly interested in decompositions whose PBBs are as reusable as possible. We note that, on average, the smaller a PBB is, the more conditions it can take part in, simply because it is more likely that all of its elements are present in a condition. Therefore, we choose decompositions with the smaller possible PBBs as a starting point for finding those with highest reusability.

Finding decompositions consisting of small PBBs is equivalent to requiring *B* to be as sparse as possible. In this work, we used the algorithm presented in [54] to find matrices *B* with few non-zero entries. In brief, the algorithm iteratively finds, for a given matrix *C*, several possible decompositions into *k* PBBs. From each of them, it generates several sparser decompositions into *k* + 1 PBBs by moving elements shared by two existing PBBs to a new PBB. This process can only go on as long as PBBs overlap, which happens if *k* ≤ *r*(*C*), the number of different rows of the matrix *C* (not to be confused with the binary rank of matrix *C*). Notice that this algorithm forces pairs of elements that are present in exactly the same conditions to also be present always in the same PBBs. For each of the decompositions output by this algorithm, we maximize its average reusability by gradient ascent. This is done by removing elements from PBBs (i.e. 1’s are removed from matrix *B*) as long as the identity *C* = *σ* (*B* *S*) holds from some matrix *S*. The elements are removed in order, starting from the one whose removal increases the average reusability of *B* the most. A Python implementation of this algorithm is available at: https://github.com/syats/ModuleReusability.

Note that the problem we are dealing with has a discrete space of solutions (binary matrices), as well as a discrete objective function (average PBB reusability). For this reason, a combinatorial algorithm such as the one chosen is more suitable than continuous methods, such as alternating gradient descent or its variations used in sparse coding algorithms (e.g. [55,56]).

We call the decomposition of a matrix *C* into *k* PBBs which are as reusable as possible a *k*-maximally reusable decomposition (*k*-MRD). The two most important features of *k*-MRDs are that they constitute a decomposition as defined above, and that the PBBs that constitute them are maximally reusable. The PBBs of a *k*-MRD can overlap among themselves, although they tend not to because maximizing reusability tends to minimize overlap. Importantly, rather than making any assumption regarding *k*, the number of PBBs, we explore the interplay between *k* and the PBB size and reusability distributions of *k*-MRDs. These distributions are different for different matrices *C*. We call the reusability of a *k*-MRD of a system the average *k*-reusability of the system.

We do not presume that any one of the PBBs constituting a *k*-MRD is functionally, evolutionarily or otherwise relevant. Rather, by finding maximum reusability decompositions we describe a property of the system as a whole: i.e. we ask how reusable are the modules the system can be decomposed into, and how are their sizes distributed. It is clear that, if modules are indeed present in the system, there might be no biological process compelling them to be of maximum reusability or minimal size. However, by finding *k*-MRDs we provide an upper bound for the average PBB reusability of any decomposition, including those in which PBBs have a biological basis, or comply with some definition of a biological module.

## Appendix B. Randomized equivalents

Given a presence/absence matrix *C* ∈ {0, 1}^*m*×*n*^, two kinds of randomized versions of it are created.

The first are density-preserving random matrices (DP-Rand). The process to create these starts with a matrix *R* of the same dimensions as *C*, whose entries are drawn from a uniform distribution between 0 and 1. Then those entries of *R* which are smaller than the density of *C* are set to 1, the rest are set to 0. A postprocessing step takes place, in which all rows and columns of *R* are checked to ensure none have zero sum; if any do, then an entry is set to 1 in it, and an entry chosen at random from *R* is set to 0. This process is repeated until all rows and columns have non-zero sum. Finally, the matrix is checked as described below for conditions contained in others; if rejected, it is discarded and another created from scratch.

The second type of matrices are RSS-preserving random matrices (RSS-Rand). These preserve the distribution of the row sum sequence in the input matrix *C*, which is a stronger condition than either preserving the per column density or preserving its row sum distribution. That is, if the original matrix has *n*_*q*_ rows with *q* ones, then the random matrix will also have *n*_*q*_ rows with *q* ones. The process to generate this random matrix starts with an empty matrix, *R*. Then, for every row index x∈{1…m}, the row sum $n_{x}=\sum_{i}C[x,i]$ is computed, and *n*_*x*_ entries of *R* are chosen at random without replacement using the function *sample* from the *random* module of Python v.2.7.^3^ These entries are set to 1 in the *x*’th row of *R*. Afterwards, the rows of *R* are shuffled, and finally the matrix is checked as described below for conditions contained in others; if rejected, it is discarded and another created from scratch.

One of the assumptions in this work is that, in the matrices being decomposed, the set of elements active in one condition cannot be a subset of those active in another. In the case of randomly generated matrices *R*, this is checked after generation by computing $RR^{T}$, and checking if its *i*_1_, *i*_2_ entry is strictly smaller than $\sum_{x}R[i_{1},x]$. If this is not the case, the matrix is discarded and a new one is generated.

For all miRNA datasets 50 DP-Rand and 50 RSS-Rand equivalents were computed, while for the EST-based protein expression data only four of each kind were computed.

## Appendix C. Alternating modular fitness functions and PBBs

Kashtan & Alon [15] introduce a model of the evolution of modularity, in which the modules found in the evolved (artificial) individuals correspond to common features in two alternating fitness functions used for selection in an artificial evolution experiment. More concretely, the population in these experiments consists of circuits made of logic gates. These circuits undergo variation by rewiring, and are evaluated for selection by matching their computed truth table to that of target logic functions. Two target functions *G*_1_ and *G*_2_ are alternated every 20 generations and the resulting best-adapted individuals can not only correctly compute both functions (a few generations after the switch), but also exhibit a modular design. This modular design consists of sets of gates that are not removed or rewired when a switch in target function occurs. In a sense, these ‘conserved’ sub-circuits are equivalent to the PBBs introduced in this work.

We posit that if the changes in target functions are drastic, then adaptations for one environment would be mostly useless for another, thus leading to low reusability of components. On the contrary, if functions *G*_1_ and *G*_2_ are similar, then large reusable modules will appear. The more similar the functions are, the larger the reused modules will be. It is in this sense that we can infer the magnitude of changes in the environment (target functions) by observing the size distribution of PBBs.
