# Supplementary material for: Reusable building blocks in biological systems
Source: J R Soc Interface. 2018 Dec 19;15(149):20180595. doi: 10.1098/rsif.2018.0595 (PMC6303794; doi:10.1098/rsif.2018.0595)
Supplement: Gene usage distribution across mammalian and chicken organs [file rsif20180595supp1.pdf]

Supplementary figures

List of Figures

1 **Supplementary Figure 1. Gene usage distribution across mammalian and chicken organs.** In [1], the authors have compiled gene expression levels for different organs in different mammalian species and chicken. Counting the number of organs in which every gene is expressed reveals a very large number of constitutive genes, and, in most cases, a slightly large number of specific genes. 2

References

[1] Brawand D, *et al.*, 2011 The evolution of gene expression levels in mammalian organs. *Nat.* **478**, 343–8. doi:10.1038/nature10532

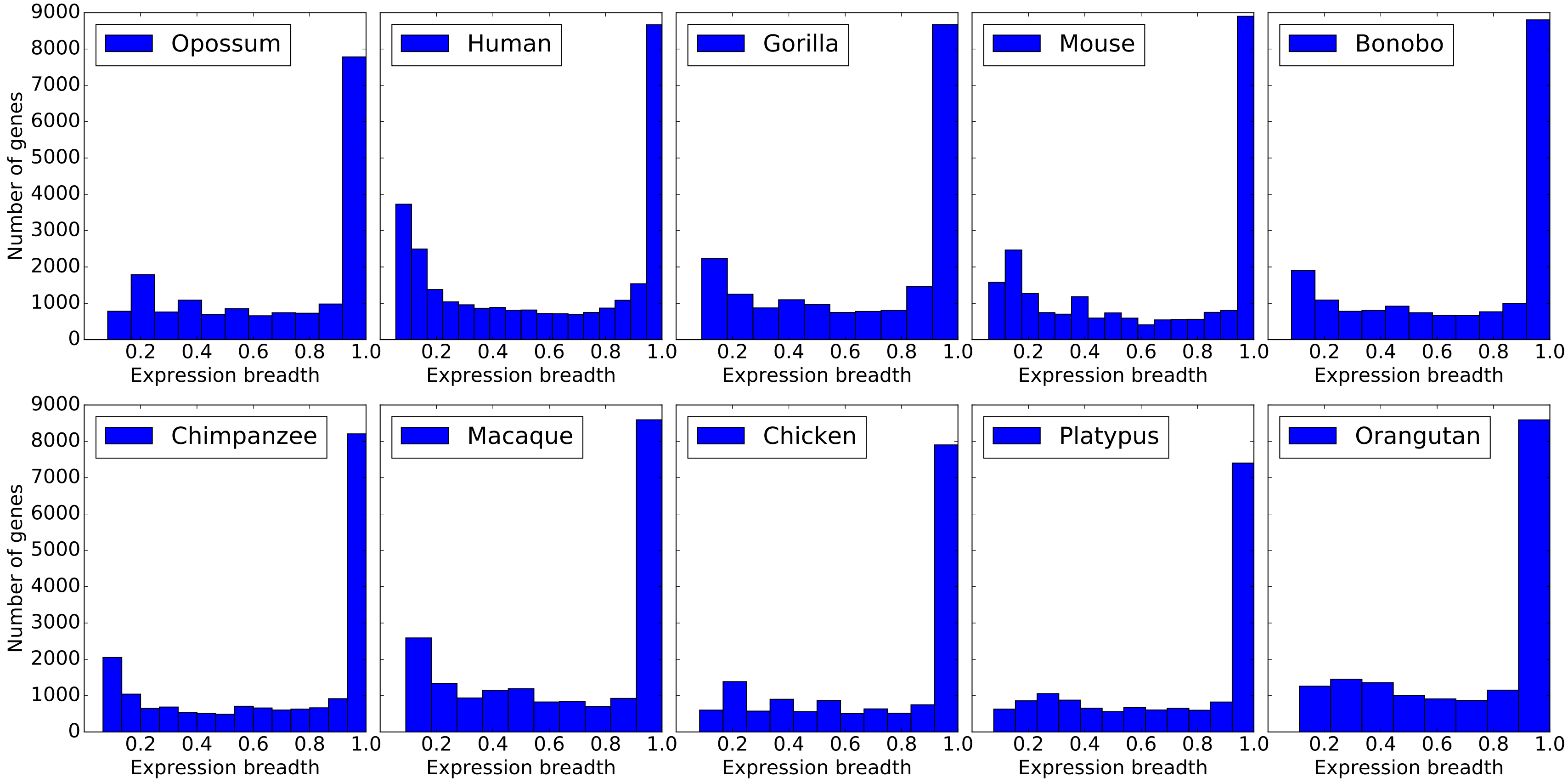

Figure 1: **Supplementary Figure 1. Gene usage distribution across mammalian and chicken organs.** In [1], the authors have compiled gene expression levels for different organs in different mammalian species and chicken. Counting the number of organs in which every gene is expressed reveals a very large number of constitutive genes, and, in most cases, a slightly large number of specific genes.

Supplementary Figure 1. Distribution of gene usage across organs, data from [1]
